# Observational research with adolescents: a framework for the management of the parental permission

**DOI:** 10.1186/1472-6939-14-2

**Published:** 2013-01-03

**Authors:** Miguel Ruiz-Canela, Cristina Lopez-del Burgo, Silvia Carlos, Maria Calatrava, Carlos Beltramo, Alfonso Osorio, Jokin de Irala

**Affiliations:** 1 Institute for Culture and Society, University of Navarra, Pamplona, Spain; 2 Department of Biomedical Humanities, University of Navarra, Pamplona, Spain; 3 Department of Preventive Medicine and Public Health, University of Navarra, Pamplona, Spain; 4 Department of Education, University of Navarra, Pamplona, Spain

**Keywords:** Adolescents, Parental consent, Research ethics, Observational research, Health surveys, Research subjects

## Abstract

**Background:**

Waiving parent permission can be an option in some epidemiological and social research with adolescents. However, exemptions have not been uniformly considered or applied. Our aim is to critically assess the different factors that could be taken into account when making decisions about waiving active parental permission in observational research with adolescents.

**Discussion:**

In some cases alternatives to parental permission could be applied to protect the rights of both adolescents and parents and also to assure the benefits to adolescents as a group that can come from appropriately conducted studies. However, the criteria of ensuring minimal risk can be difficult to define and apply and a distinction between harm and discomfort is reviewed. Waiving active parental permission could be acceptable when the risk of harm is minimal; when the research questions are related to an activity for which adolescents are not legally considered to be children; when the risk of harm or discomfort may increase if parental permission is required; and when risk of discomfort is low because the questionnaire is not potentially offensive for some adolescents and/or for some parents.

**Summary:**

Stringent rules concerning parental permission in some studies could be detrimental to adolescents. A framework and a decision tree guide are proposed to help researchers and Research Ethics Committees in their decisions on whether active parental permission must be obtained.

## Background

The relationship between ethics and epidemiology has been explored for many years [1]. International guidelines have been published to elucidate the ethical rules that should govern epidemiological research [2]. Generally, fewer ethical issues are raised in observational studies than in experimental studies [3]. However, there is challenging tension in observational research between the need to access personal information and the need to maintain due respect to participants’ autonomy and privacy [4-7]. Requiring informed consent is a contentious issue because in some cases it makes it very difficult, if not impossible, to carry out epidemiological research [6,8]. Allowing some possible exemptions to the principle of explicit consent is currently being debated [9,10].

The debate concerning exemption for parental permission is especially contentious issue in epidemiological research with adolescents. The use of the term “parental permission” rather than “parental informed consent” is preferable because parents or legal guardians are not the research subjects and they do not bear the risks and benefits of participation [11,12]. However, both terms are used interchangeably in literature. In any case, the generally accepted rule is to obtain both the minor’s assent and parental (or legal representative) permission before conducting any research [2]. This is an essential requirement in clinical trials with minors [13]. However, waiving parent permission can be an option in some epidemiological and social research with adolescents where risks are considered minimal [2,11]. This waiver has been justified in non-interventional studies conducted in health behavior [14], social [15] and psychological research [16].

The current problem is that exemptions are not uniformly considered or applied. In some countries there is no clear set of rules regarding when it is required to obtain parental permission for minors in observational research [17]. Regulation in the US allows the consideration of a waiver of parental permission when it is not a reasonable requirement to protect subjects. However, even in these cases there is some controversy and contradictory assessments from Research Ethics Committees can be found [18-20]. This variability can be explained by multiple factors that can determine decisions regarding adolescents’ participation in research. In each case the assessment should take into account the topic of the research, the context in which it is implemented and the developmental level and age of the participants [21].

**Figure 1:**
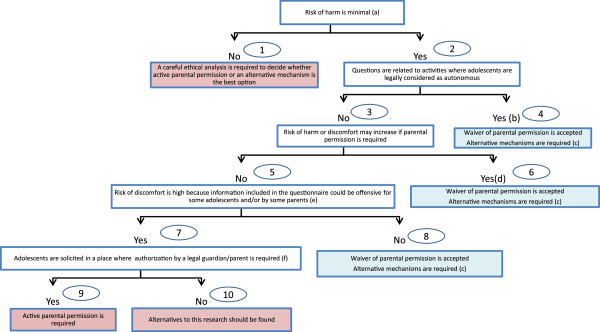
Decision tree guide regarding parental permission in research with adolescents*. * Numbers in circles enable the reader of the text to follow the explanation on this figure. **(a)** Harm is an objective measure related to the risk of disclosure of the information obtained in the study. Anonymous studies or studies adhered to standards of data security are usually considered to be minimal risk. However, information could promote risky behaviors depending on the age of the adolescents and/or their level of maturity. **(b)** When the legal autonomy of adolescents or a particular research topic is controversial, the level of discomfort related to the research should be assessed. **(c)** A variety of alternatives can be used, such as passive parental consent, permission from a surrogate parent or child’s advocate, and consultation with key school/community members. **(d) **Extreme cases, such as neglected or abused children, where risks could be exacerbated if parents know the participation of the adolescent. **(e)** For example, the school board may consider that, due to the nature of some information included in the questionnaire or the aim of the research, it could be inappropriate to leave the decision making in the hands of the school rather than the parents.** (f) **This question refers to the context of the research. Adolescents in the school are always under the guardianship of their parents and they are easily accessible (9) but this situation is less clear in community-based research or in the Internet (10).

To our knowledge, the current literature does not provide a specific and detailed guide about how to manage parental permission in different research scenarios. The Guidelines for Adolescent Health Research made a significant contribution to this question but mainly taking into account the US legal context [11]. In most published studies a waiver of parental permission is allowed on specific, and in some cases, exceptional cases that make it difficult to know if it can be extrapolated to other type of research. Our aim is to critically assess the different factors that should be taken into account when making decisions about a waiver of parental or legal guardian permission in observational research with adolescents. A framework and a decision tree guide (Figure 1) are proposed to help researchers and Institutional Review Boards (or Research Ethics Committees) in their decisions on whether parental permission must be obtained or whether other alternatives to protect adolescents should be considered.

## Discussion

### Vulnerability and respect for adolescents

The principle of respect for persons stated at the Belmont Report means that individuals should be treated as autonomous agents and that persons with diminished autonomy are entitled to protection [22]. A balance of both ethical mandates needs to be found in the case of research with adolescents. When assessing the nature and level of protection needed for adolescents, the focus should be on the specific circumstances that might contribute to vulnerability rather than only the definition of the participating group [11]. Following guidelines on clinical research with minors (where risk is frequently greater than minimal) the general rule is that parental permission must be obtained. However, in research related to a health service where adolescents are considered legally autonomous (for example, a clinical trial of a new drug for herpes virus using adolescents from a sexually transmitted infection clinic) some may argue that this research could be conducted without active parental permission. These situations are beyond the scope of this paper and a careful ethical analysis must be made of this kind of research (Figure 1, No. 1). When the risk is minimal, the required levels of parental involvement should be determined after evaluating several factors such as the age and the capacity of adolescents to make decisions.

#### The evolving capacities of adolescents

As stated before, the general approach in today’s research environment is to treat adolescents as children and to ask for parental permission in research. However, in some cases this could be described as a protectionist and paternalistic policy [14]. Even if adolescents are legally considered children (Figure 1, No. 3), investigators and Research Ethics Committees or IRBs may still decide that waiving parental permission is the best option to protect adolescents and to allow research which entails minimal risk (Figure 1, Nos. 6 & 8) [11].

Article 5 of the UN Convention on the Rights of the Child states that the responsibilities, rights and duties of parents should be respected in a manner consistent with the evolving capacities of the child [23]. Article 12 of this Convention states that the views of the child should being given “due weight in accordance with the age and maturity of the child”. The National Commission in the US noted that parental permission would not be required on research in which the subjects are “mature minors” and the procedures involved entail essentially no more than minimal risk that such individuals might reasonably assume on their own (Figure 1, Nos. 6 & 8).

The World Health Organization defines adolescence as the period between 10 to 19 years of age [24]. Different authors and organizations have proposed several ways of dividing this period. Table 1 shows four adolescent development stages according to age and sex [25]. During these stages adolescents experience multiple biological, cognitive and socio-emotional changes. Early adolescence is the period where adolescents develop the potential for more abstract types of thinking and begin to explore the world with more independence and less parental supervision [25]. Decision-making abilities develop during the early adolescence although genuine increases in behavioral autonomy occur in middle and late adolescence (between ages 14/15 and 18).

**Table 1:** Adolescent stages

	***Preadolescence***	***Early Adolescence***	***Middle Adolescence***	***Late Adolescence***
Girls	9-12 yrs.	12-14 yrs.	14-16 yrs.	16-18 yrs.
Boys	10-13 yrs.	13-15 yrs.	15-17 yrs.	17-18 yrs.

Research policies usually do not address the rapidly changing competencies of adolescents from 12 to 18 [21]. A review found that the crossover age in understanding information in medical research was between 7 and 11 years of age [26]. Bruzzese and Fisher found that youth between 10 and 15 were close, but didn’t have the same levels of understanding and exerting their research rights as adults [27]. Several studies have demonstrated that adolescents as young as 14 are competent to make informed decisions concerning their health [28] and it is suggested that around the age of 15 adolescents become eligible for the classification of mature minors [29]. A study carried out in the US found that 14 year-olds demonstrated a level of competence equivalent to that of adults according to legal standards when presented with hypothetical treatment dilemmas [30]. The Guidelines for Adolescent Health Research state that “for mid- and late adolescents (aged 14 years old or older), understanding of research and cognitive ability to make decisions about research participation are similar to these abilities in adults” [11].

#### Adolescents who are considered legally as autonomous

Adolescents are recognized as having an increased capacity to make independent decisions concerning their own health. In many countries they can give permission for sensitive health services, such as HIV testing, without parental notification. Following US legislation, adolescents who have reached the age to consent for general health care, who are allowed to give their consent for specific types of health care or who are emancipated minors, are not considered children, even if they are under the legal age (the concrete legal age depends on the state) [19]. In the UK a child under 16 is allowed to consent to treatment without the permission of their parent or guardian providing that some criteria of competence are fulfilled: they have been counseled and do not wish to involve their parents; and they have sufficient maturity [15]. In Denmark adolescents (from the age of 12 onward) have a certain level of self-determination on health, social and educational contexts [17].

The legal capacity of adolescents to make independent decisions could also be applied to the research context. The National Commission in the US stated that IRBs may determine that parental permission is not appropriate in “research designed to identify factors related to the incidence or treatment of certain conditions in adolescents for which they may legally receive treatment without parental consent”. However, this application of the right of self-determination at the research level is usually ambiguous. A survey in the US concerning consent for adolescents showed that, under the same federal regulation, there was a wide spectrum of interpretation from IRBs, even in scenarios that included emancipated or mature minors [31]. In the UK, the Central Office for Research Ethics Committees considered that there is a profound difference between accessing medical interventions and participating in research. This organization argues that parental consent is an ethical, if not a legal, requirement prior to the involvement of minors in research [15].

However, this distinction between the ability of a young person to consent for a health care intervention and for a research related to this health care may seem contradictory in some cases [15]. For example, adolescents have the capacity to decide to have sex when they are 16 years old but parental consent would be required in an anonymous research on their sexual behavior [16]. This contradiction could be more obvious in some public health activities, such as public health surveillance, where the distinction between research and practice is mainly based on the intention for which the activity is designed [32]. We find this could be considered an unjustified inconsistency because youths’ autonomy in research should be respected if they have the capacity to consent [28]. This distinction may be arbitrary especially when research risks are lower than risks related to the healthcare services they can legally receive, or activities they can legally decide upon, without parental permission. Consequently, waiving parental permission could be accepted when risk of harm is minimal and questions included in research are related to an activity for which adolescents are not considered legally as children (Figure 1, No. 4).

However, even when adolescents are legally not considered as children and they can autonomously receive health services, parental permission should not be automatically waived. In some cases parents may believe that their responsibilities to decide on interventions that can affect the wellbeing of their children are taken away by some laws and strongly disagree with them [33,34]. The autonomy of adolescents is thus questioned by these parents. This may be a reason for why such laws concerning parental consent on controversial issues related to adolescents are not uniformly applied, even among different regions of a same country. For example, in the US only a few states do not require parental permission in a minor’s abortion [35]. In other cases, the autonomy of adolescents for a particular health service is more widely accepted, for example, healthcare policies concerning sexually transmitted infections. However, a specific research topic on adolescents using this particular health service could be controversial. For these reasons, when the legal autonomy of adolescents or a particular research topic on adolescents is controversial, we propose to additionally assess the level of discomfort related to the research (as it is developed later in this text) instead of just accepting alternatives to parental permission (Figure 1, No. 4).

### A matter of justice

Access to personally identifiable data is frequently necessary in epidemiological studies in order to answer publicly valuable research questions. In some circumstances, waiving informed consent has been justified to make this research possible or to avoid a likely bias [36,37]. The same logic is present in the case of observational research with adolescents. Parental permission can be an important source of bias if those adolescents at risk are systematically excluded because their parents deny their participation [38]. Chartier *et al*. found that a significantly lower proportion of students participated when informed consent from parents was required in a depression screening program implemented in the context of a research study [12]. Moreover, they found a differential exclusion of high-risk students when active parental permission was required. As a consequence, another relevant reason to waive parental consent is that young people are often excluded from participating in research and initiatives that may serve to improve their health [28].

Moreover, adolescents who assent to participate in research studies may be less likely to share personal and sensitive information if they suspect that their responses can be disclosed to their parents [39]. A study of adolescents 12-17 years old showed that fewer adolescents would report suicidal thoughts when told that researchers will share information with parents (1%) than when a promise of confidentiality was required (8%) [20]. This possible bias can be minimized in some studies where responses are anonymous and efforts are made to increase adolescents’ perception of anonymity [40]. However, response rates can still be low because adolescents may claim that they prefer to sign their own consent when personal and intimate issues are included in a questionnaire. This reason explained the low response rate (19%) in an anonymous study to understand factors related to the use of condoms among Brazilian adolescents students [41]. A selection bias was probably present in this study because female and public schools students were more likely to return the consent by the legal guardian and consequently they were overrepresented in the study.

Many research questions are currently unresolved because there is an insufficient number of studies with adolescents. In order to achieve a benefit for all youth, it is imperative to obtain better knowledge to be applied in health promotion initiatives for adolescents and public health policies [25]. Participation in research can be therefore a matter of justice. Denying adolescent participation because of the added complexities of determining if parental consent is necessary could be considered unfair [21]. Moreover, completing surveys may also have some benefits for adolescents who participate in this research. Participants in research may receive important information related to their own health and lifestyle. This information and the interaction with researchers may also increase their self-understanding of the risk of one’s own behavior [11].

### Risks and the protection of adolescent’s privacy

A general condition for waiving informed consent in the US is that the research involves no more than minimal risk to the subjects. Traditionally, the US regulation has defined minimal risk as when “the probability and magnitude of harm or discomfort anticipated in the research are not greater in and of themselves than those ordinarily encountered in daily life or during the performance of routine physical or psychological examinations or tests” (Common Rule 45 CFR 46.102(i)). The European Union Working Group, which provided guidance for applying the European Clinical Trials Directive with regard to research in children, adopted the same definition [42].

A substantial difference between experimental and observational research with adolescents is generally that risks in the latter are mainly related to the information obtained in the study. These risks can be bidirectional because information can have a negative impact both when adolescents are exposed to sensitive questions and when their answers to these questions are known by their parents or other persons. Confidential or anonymous survey research is considered low-risk research by the Guidelines for Adolescent Health Research. In this case, adolescent’s capacity to give consent can be assumed based on the reasonable expectation of capacity of the group of adolescents to be studied [11]. To be classified as minimal risk research, the survey should include questions about issues that are concordant with the age, family, social, and cultural characteristics of adolescents. Moreover, for some specific topics it would be appropriate to assess the intellectual and emotional development of adolescents since this development is determined by other factors than age. In the assessment of these risks, researchers could seek external advice from experts, parents, etc. with close and practical knowledge of adolescents.

The application of “minimal risk” can be difficult to concretize and a distinction between risk of harm and risk of discomfort has been proposed for its application in paediatric research in the clinical context [43]. The same reasoning can be applied in the case of observational research that is frequently implemented in epidemiology and sociology. Both harm and discomfort can be psychological or physical. However, harm is more objective because it is directly a result of the study and usually all the adolescents participating in the study are equally affected. This can occur, for example, when information the participants end up receiving by reading a questionnaire is not appropriate for their age, when the data they provide is not adequately protected, or when disclosure to parents can worsen the situation of children abused by them or close family members.

US regulation explicitly mentions neglected or abused children as a subject population for which a waiver of parental consent might be appropriate (Figure 1, No. 3). For example, Stablein and Jacobs obtained a waiver of parental consent granted by the local IRB in an ethnographic study of street youth in a city in the northeastern US. They stated that “the waiver for non-homeless youth was deemed justifiable because discussing participation with caregivers could exacerbate existing tensions in the home related to the minor’s engagement in delinquent activities on the street” [29]. Disclosure of suicidal thoughts by adolescents may increase their risk of harm when it is their home situation what has triggered their emotional stress [20]. In these extreme cases, the disclosure of information to parents could be waived in order to avoid a higher risk of harm or discomfort to adolescents (Figure 1, No. 6).

Research with adolescents may address sensitive issues. Topics may relate to an illegal behavior, such as substance abuse, or in other cases may be perceived as inappropriate (e.g. sexual intercourse). For some youth, their desire to participate in sexual health research may be interpreted as admitting to being sexually active and/or having accessed sexual health services [28]. In these cases harm may result from a loss of confidentiality and disclosure of information to others. Interestingly, the challenge is that in these cases parental consent can increase risk, at least it may be thus perceived by adolescents [18]. This reasoning was even extended to an Internet-based research project where authors thought that parental permission would not probably contribute to increase the protection or safety of participants and it would likely decrease adolescent participation [44].

Apart from the risk of harm, the level of discomfort must also be assessed in order to protect adolescents (Figure 1, No. 5). Discomfort concerns a subjective and momentary experience. The information provided in the research (for example related to sex, drugs, violence, etc.) might have a negative impact on adolescents. This information may be a source of discomfort even though it refers to activities that can be considered as normal for their age, and which they may have experienced or seen in their peers. In many studies the likelihood of discomfort occurring is relatively higher than harm because a variety of factors (cultural, religious, etc.) may be related to the subjective impact of the information on adolescents. Waiver of parental permission could be accepted in those cases where risk of discomfort is low (Figure 1, No. 8). If risks are high, the decision should depend on whether adolescents are solicited in a place where authorization from parents or legal guardians is available (Figure 1, No. 7).

A source of concern is that by asking adolescents sensitive questions regarding drug use, sexual behavior, or violent activities, those behaviors could be perceived as justified and adolescents would be wrongfully influenced. However, some authors argue that there is no literature to support this and there is no reason to think that providing some information to adolescents will change their behavior, especially when well-designed health promotion programs have produced modest changes [14]. We believe that the influence on behavior probably depends on what questions are asked and how they are asked. Another source of concern, especially for parents, is that information could be offensive (risk of discomfort). In these situations the benefits of parental preferences might potentially be offset by adolescents’ well-being resulting from the research [12]. This well-being refers both to the respect for their own autonomy and to the benefits that may be obtained by participating in the research.

Finally, it should be taken into account that this debate is not limited exclusively to observational research since there are experimental designs with similarly minimal risks. For example, an experimental study with 1,588 youth (age 16-25 years) assessed a sexual health education programme delivered via a Facebook page [44]. The IRB approved a waiver of parental permission in this study and the authors stated that risks were minimal because minors were already allowed to consent for sexually transmitted infection testing without parental permission. This example illustrates the necessity of determining ethical assessment criteria for research based on its level of risk relative to information, rather than on the type of design (experimental vs. observational).

### Alternative mechanisms to protect adolescents

Apart from the assessment of risks, US regulation allows the parental permission requirement to be waived when “the subject population is one where parental or guardian permission is not a reasonable requirement to protect the subjects, provided there is an appropriate substitute mechanism for their protection” (Federal Regulations 45 CFR § 46.408(c)). The interpretation of this rule is not clear but a general conclusion could be that alternative mechanisms should be accepted when there are better ways to respect adolescents and to allow research. A variety of alternatives can be found such as passive parental consent, appointment of a child advocate who is unconnected with the research project, permission from a surrogate parent (such as a social worker, school staff, nurse or physician), guidance by any adult other than parents and consultation with key community members [11,45]. The use of one of these alternatives will depend on the context of the research. A key aspect to determining whether the authorization of a legal guardian is required is where adolescents are solicited to participate in the research (Figure 1, No. 7). We will focus on two main scenarios, school-based and community-based research.

#### School- based research

In general, schools should take parents into account when dealing with issues related to their children’s wellbeing. However, active parental permission is not necessarily the best option in all research taking place in schools. There are a number of cases where there can be better alternatives. Active permission implies that a signed form must be returned to the school to indicate the parents’ approval. Several studies have shown that this type of consent is related to a decline in the response rate and that it can also be a potential source of bias [12,46-48]. Other authors have shown that it is possible to achieve a high response rate with active consent in school-based research [40,49]. However, this requires implementing multiple strategies with the participation of school principals, teachers, parents and students [50,51]. The resources and time spent on implementing these strategies should be proportionate to the risk and benefits of the research.

The US Congress introduced the Protection of Pupil Rights Act in 1978 to address the vast number of surveys and psychological tests that were being administered within public elementary and secondary schools. One of the original purposes was to obtain active parental consent. However, several court decisions have shown that this act lacks an enforcement mechanism. A court declared that, once the children arrive at a public school, parents lose their voice in controlling what their children are exposed to [52]. This statement comes from a case relating to a survey among first, third, and fifth grade students in California. Parents received a request for their permission but were not informed that the survey included sexually-explicit questions. Several parents complained but the district court dismissed the suit, and the parents appealed to the Ninth Circuit, which confirmed the lower court's dismissal. This court’s final decision left no redress for parents other than removing their children from the public school system, petitioning the legislature, or electing a new school board [53].

With respect to this case, there is a shared authority between parents and the state in public schools [54]. Public school educators were acting in the place of parents (*in loco parentis*) [14]. This situation is similar in private schools because there is also a shared responsibility among parents and teachers in the education of adolescents. School authorities have a right and shared a responsibility “to determine the sexual awareness of their students so that they can adopt curricula that teaches healthy ways of dealing with sexual language, information, and impulses” [55]. This case cannot be extrapolated to other countries where all research depends on their specific legislation. However, it is likely that in many cases there will be a lack of regulation or at least an ambiguous application of more general rules. Communication and cooperation between parents and the school are key aspects of solving possible conflicts [52].

Any decision about whether to require active parental permission should weigh the parents’ perspectives and responsibilities, the children’s ability to comprehend and make a free choice without coercion, and the possibility of systematic exclusion of students [12]. The amount of resources and time required should also be taken into account. However, waiving active parental permission should be allowed only when research is considered an activity that it is part of the shared responsibility recognized by the parents and the school. It is critical to determine the risks of discomfort for students. Active permission from parents should be required if these risks are considered high (Figure 1, No. 9).

In low-risk cases, alternative mechanisms could be a better option. A report on research governance and ethics in children’s services, commissioned by the Department of Education in England, stated that in some circumstances it may not be appropriate to seek adult permission for research with children [56]. This report also indicated that there are some exceptional circumstances where adult gatekeepers (including parents or professionals charged with caring for children) should not be informed if a child is going to be approached as a potential participant. A group of Danish researchers argued that adolescents 15 or older should have the right to make an independent decision as to whether or not they want to participate in a study dealing with issues that are relevant to them, always assuming that the relevant authorities and school boards have approved the study [17].

In general, the involvement of parents and other community representatives on advisory boards is relevant since these boards can communicate concerns to researchers and advise on the most appropriate and effective strategy [11]. However, “passive consent” from parents is an opt-out procedure which has been frequently used in school surveys as an alternative to active permission. In this case, parents receive information about the study and a non-response is assumed to be an acceptance to participate. The same type of permission is obtained for parents in order to involve their children in extracurricular activities organized by the school. This procedure does not enable us to know whether parents have read the information and if they have really consented. However, a modified protocol requiring an acknowledgment of receipt could be implemented; for example, a confirmation that an email explaining the research has been received or sending the information via certified mail. For this reason, the use of “passive consent” in schools could be considered a good alternative when risks of discomfort are not high (Figure 1, No. 8). Discomfort should be low when questions included in the study are appropriate for the students’ development and if they would be acceptable for most of the parents. This could be reviewed with school boards where parents are involved.

#### Community-based research

Much epidemiological and social science research is community-based. Adolescents are invited to participate in research when they are in a center which offers a social or health services, such as sexual health, when they are in the streets, or through the Internet. Research risks can be considered minimal if the information obtained is appropriate to their age. The key aspect in these situations is to decide whether an alternative mechanism to parental permission is adequate in order to protect adolescents’ best interest. An alternative is to obtain permission from a surrogate parent; however, in many cases it will not be possible to clearly identify the adequate person. For this reason a better alternative can be to obtain the approval by adolescents’ advocates or key community members.

A good example is the Toronto Teen Survey, research undertaken by Planned Parenthood of Toronto with around 1,200 teenagers (ages 13-17) in a community-based service setting. The goal was to identify access barriers and facilitators to community sexual health resources. The ethics review board allowed parental permission to be waived [28]. The informed consent process was supported by youth advisory committees and their opinion was that youths were more likely to respond honestly to surveys given out and explained by their peers in community settings rather than in school or at home. The waiver of consent was also supported by the youth advisory committees because they considered that mandating parental consent would significantly limit participation.

Third party legal advocates have also been proposed as an alternative to parental permission in cases of abuse and neglect and for other at-risk youth populations [29]. The approach to adolescents in these cases is usually outside their homes or the school. Some studies, mostly qualitative, need to be implemented in places where adolescents are developing risky activities. In other cases young participants are approached on the Internet, for example, to learn about their life-style through their participation in social networking. In these situations it is not possible to know whether adolescents’ participation in activities carried out in the streets or on the Internet has parental or legal guardian approval.

The best ethical approach in this case is to allow the waiver of parental permission only when risks of discomfort are not high (Figure 1, No. 8). This can be established if the information obtained in the study is related to activities that adolescents are already undertaking in the context where research is being conducted. In these situations, the best alternative mechanism to protect the best interest of adolescents could be to obtain the guidance and authorization of key community members. Taking into account the difficulties of accessing parents, alternative methods of research should be found if risks of discomfort are estimated to be high (Figure 1, No. 10).

## Summary

A rigid requirement to always gain parental permission may end up doing more harm than good. The protection of adolescents should take several factors into account such as the research topic, where it is implemented, their age and the extent to which these factors affect their vulnerability. Risks related to observational research are mainly related to the information obtained from and/or given to adolescents. The protection of adolescents’ confidentiality and the respect of their autonomy are two relevant factors to weigh when deciding about whether to ask for parental permission. The urgent need to solve health and social problems specific to adolescents could make it unjust to prohibit some research only because of the requirement to obtain parental permission. For all these reasons, in some cases there will be better options to respect adolescents’ interest such as passive parental permission, permission from a surrogate parent, and consultation and guidance of key community members. Both researchers and Research Ethics Committees should decide in each case according to the best direct interest of the adolescents and to the indirect benefit that the research results may have for them as a group.

## Competing interest

The authors have no conflict of interests to disclose. This study is part of a research project called *YOURLIFE*, partially funded by the Institute for Culture and Society (University of Navarra).

## Authors’ contributions

MRC, CB, and JI conceptualized the idea of the study. MRC, AO, and JI performed the literature review and wrote the first draft of the manuscript. AO, CLB, SC, MC and CB reviewed and revised drafts of the manuscript and the figure. All authors approved the final version of the submitted manuscript.

## Pre-publication history

The pre-publication history for this paper can be accessed here:

http://www.biomedcentral.com/1472-6939/14/2/prepub
